# P-408. Utility of a Large Language Model for Identifying Central Line-Associated Bloodstream Infections (CLABSI) Using Real Clinical Data at Stanford Health Care

**DOI:** 10.1093/ofid/ofae631.609

**Published:** 2025-01-29

**Authors:** Guillermo Rodriguez-Nava, Goar Egoryan, Katherine E Goodman, Daniel J Morgan, Jorge Salinas

**Affiliations:** Stanford University School of Medicine, Stanford, CA; Stanford University, Menlo Park, California; University of Maryland School of Medicine, Baltimore, Maryland; University of Maryland School of Medicine, Baltimore, Maryland; Stanford University, Menlo Park, California

## Abstract

**Background:**

Central line-associated bloodstream infections (CLABSI) surveillance can be subjective and time-consuming. Large language models (LLMs) are advanced artificial intelligence systems with potential to assist healthcare professionals in classification tasks. Stanford Health Care recently implemented one of the first secure LLMs, powered by OpenAI’s GPT 4.0, cleared for sensitive health data. We assessed its performance in classifying CLABSI cases.

**Figure 1:**
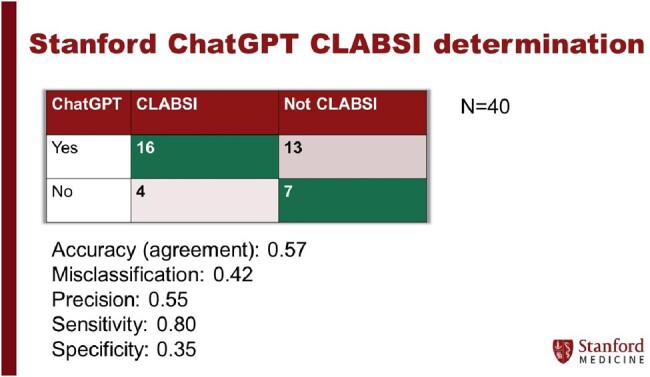
Confusion Matrix of LLM Performance in CLABSI Classification.

**Methods:**

We selected 40 patients flagged by our surveillance system for CLABSI review from November 2023–March 2024: 20 CLABSIs, consecutively identified, and 20 not-CLABSIs (randomly sampled). We prompted the LLM to determine if patients met the NHSN definition for CLABSI and provided the blood culture results that triggered the alert and the last 2 progress notes from the primary care team at the infection window end (within 3 days after the first positive test). We compared the secure LLM's determinations with those of infection preventionists.

**Table 1.**
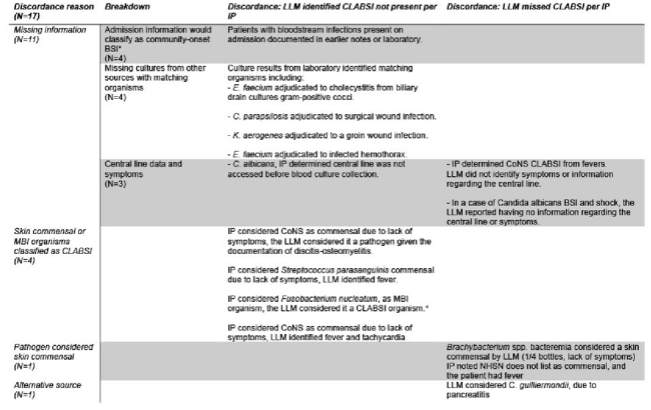
Cases in which the LLM did not agree with IP assessment for CLABSI. *Community-onset: Blood cultures obtained within 2 days of admission. +NHSN guidelines list Fusobacterium nucleatum as an MBI organism (https://www.cdc.gov/nhsn/pdfs/pscmanual/17pscnosinfdef_current.pdf) Abbreviations: BSI, bloodstream infection; CLABSI, central line-associated infection; CoNS, coagulase-negative Staphylococci; ESBL, extended-spectrum beta lactamase; HIDA scan, IP, infection preventionist, LLM, large language model; MBI, mucosal barrier injury; MSSA, methicillin-susceptible Staphylococcus aureus; NHSN, National Healthcare Safety Network.

**Results:**

Across 20 CLABSI-positive and 20 CLABSI-negative cases reviewed, the LLM accurately identified 16 of 20 CLABSIs and 7 of 20 not CLABSIs. The sensitivity was 80% (95% CI 57.6%–92.9%), specificity was 35% (95% CI 33.3%–86.5%), and the agreement rate was 57.5% (95% CI 41.2%–73.3%). Among 17 discordant cases, 11 involved clinical data available in the chart but unavailable to the LLM—admission information (4 false-positives), matching organisms (4 false-positives), and central line or symptom status (2 false-negatives, 1 false-positive). If this information was available to the LLM, we expect an adjusted sensitivity of 90% (18/20) and adjusted specificity of 80% (16/20). The remaining discordant cases involved misclassifications of organisms and incorrect identification of infection sources by the LLM. The mean review time by infection preventionists was 75 minutes (SD 48.7 minutes) compared to 5 minutes using the LLM.

**Conclusion:**

An LLM not specifically trained for CLABSI classification showed high sensitivity using limited patient data. LLM case review required 5 minutes, versus 1 hour for traditional review. These results suggest LLMs could serve as a "first-pass" screening tool for CLABSI detection, helping infection preventionists narrow records needing human review.

**Disclosures:**

**All Authors**: No reported disclosures

